# Dissecting the contributions of membrane affinity and bivalency of the spider venom protein DkTx to its sustained mode of TRPV1 activation

**DOI:** 10.1016/j.jbc.2023.104903

**Published:** 2023-06-10

**Authors:** Yashaswi Singh, Debayan Sarkar, Subhadeep Duari, Shashaank G, Pawas Kumar Indra Guru, Hrishikesh M V, Dheerendra Singh, Sahil Bhardwaj, Jeet Kalia

**Affiliations:** 1 Department of Biology, Indian Institute of Science Education and Research (IISER) Pune, Pune, Maharashtra, India; 2 Department of Biological Sciences, Indian Institute of Science Education and Research (IISER) Bhopal, Bhopal, Madhya Pradesh, India; 3 Department of Chemistry, Indian Institute of Science Education and Research (IISER) Pune, Pune, Maharashtra, India; 4 Department of Chemistry, Indian Institute of Science Education and Research (IISER) Bhopal, Bhopal, Madhya Pradesh, India

**Keywords:** bivalency, DkTx, ICK toxin, Kv channels, membrane partitioning, pain, protein-lipid interactions, sortase ligation, spider toxins, TRP channels, TRPV1, valence

## Abstract

The spider venom protein, double-knot toxin (DkTx), partitions into the cellular membrane and binds bivalently to the pain-sensing ion channel, TRPV1, triggering long-lasting channel activation. In contrast, its monovalent single knots membrane partition poorly and invoke rapidly reversible TRPV1 activation. To discern the contributions of the bivalency and membrane affinity of DkTx to its sustained mode of action, here, we developed diverse toxin variants including those containing truncated linkers between individual knots, precluding bivalent binding. Additionally, by appending the single-knot domains to the Kv2.1 channel-targeting toxin, SGTx, we created monovalent double-knot proteins that demonstrated higher membrane affinity and more sustained TRPV1 activation than the single-knots. We also produced hyper-membrane affinity-possessing tetra-knot proteins, (DkTx)_2_ and DkTx-(SGTx)_2_, that demonstrated longer-lasting TRPV1 activation than DkTx, establishing the central role of the membrane affinity of DkTx in endowing it with its sustained TRPV1 activation properties. These results suggest that high membrane affinity-possessing TRPV1 agonists can potentially serve as long-acting analgesics.

Polyvalency enables the creation of tight biomolecular complexes (1, 2, 3). This phenomenon has been extensively studied on immunoglobulin (IgG) proteins that bind bivalently to antigens (4, 5). Bivalent binding has been demonstrated to endow IgG–antigen complexes with dissociation constant values that are two to three orders of magnitude lower as compared to monovalent binding (6, 7, 8, 9).

Bivalency has been recently discovered to be employed by animal venom toxins to bind to their target ion channel proteins (10, 11). The founding member of the family of ion channel-targeting bivalent toxins is the double-knot toxin (DkTx) (10), a spider toxin that potently activates the cation-selective homotetrameric transient receptor potential vanilloid 1 (TRPV1) ion channel (12, 13). DkTx contains two cysteine-rich lobes (the K1 and K2 knots) connected to each other *via* a seven amino acids-long linker (Fig. 1*A*). These lobes belong to the inhibitor cystine-knot (ICK) family of toxins, a well-known class of ion channel-modulatory peptides expressed in the venom of poisonous animals such as spiders and snakes (14, 15). Functional, structural, and molecular dynamics simulation-based studies reveal that DkTx partitions into the membrane and that its two knots bind to the outer pore region of TRPV1 (Fig. 1*A*) causing sustained channel activation (10, 16, 17).

**Figure 1:**
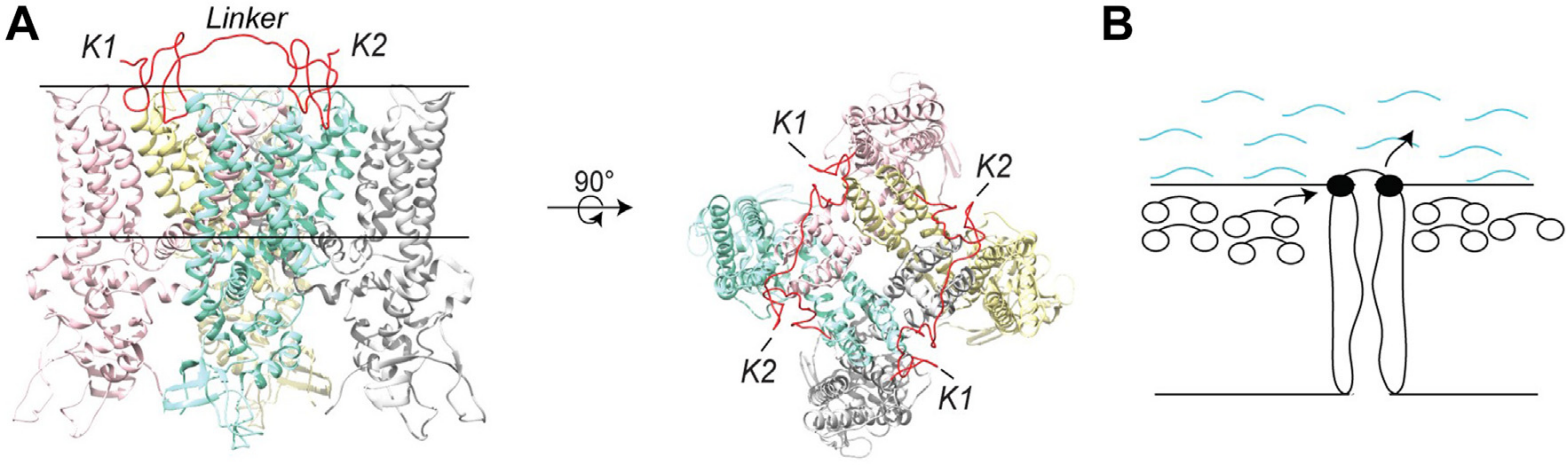
**The DkTx-TRPV1 system.***A*, the DkTx-TRPV1 complex (PDB: 5irx) (16) viewed sideways from within the membrane (*left*), and extracellularly, from the *top* (*right*). For the sake of clarity, only one DkTx molecule out of the two present in the structure is depicted in the *left panel*, and the disulfide linkages have been omitted. *B*, the toxin relay hypothesis. DkTx molecules accumulated within the membrane are depicted as dumbbell-shaped objects with their two knots represented as *white ovals*, and the DkTx molecule bound to the channel is depicted as a dumbbell with *black ovals*. Only two subunits of the TRPV1 tetramer are depicted for clarity. The wavy *blue lines* indicate the extracellular solution.

The single K1 and K2 knots can individually activate TRPV1 albeit with much poorer potency and considerably faster wash-off rates vis-à-vis DkTx (10, 17). Considering the well-established augmentation of the binding affinity of ligand–receptor complexes due to bivalency as in IgG-antigen complexes alluded to above, not surprisingly, the much slower wash-off rates of DkTx as compared to the single-knots have been attributed to its bivalency (10). More recently, the high membrane affinity of DkTx has also been implicated in endowing it with its sustained mode of action based on the finding that the wash-off rates of site-directed DkTx variants are negatively correlated with their membrane affinities (18). Based on this observation, a model was proposed that hypothesized that the high membrane partitioning ability of DkTx enables it to accumulate at elevated concentrations in the membrane so that when the channel-bound toxin molecules are dislodged during wash-off, they are efficiently replaced by toxin molecules in the membrane resulting in the establishment of a “toxin relay” leading to sustained channel activation (Fig. 1*B*) (18). Notably, this hypothesis is also supported by the observation that the fast washing-off K1 and K2 single-knots partition poorly into membranes (17). Considering that the single-knots are monovalent as well as demonstrate poor membrane partitioning propensity, it is not clear whether they wash-off fast because they lack bivalency or/and due to their poor membrane affinities.

Herein, we investigate the contributions of the bivalency and membrane affinity of DkTx to its TRPV1 activation properties by generating and characterizing a series of DkTx variants possessing a range of valences and membrane affinities. Our results demonstrate that the high membrane affinity of DkTx is a key determinant of its sustained mode of TRPV1 activation. Considering that TRPV1 plays a central role in the pain-sensing pathway and that its agonists such as capsaicin and resiniferatoxin are used clinically as analgesics (19, 20), our results suggest that TRPV1 agonists possessing high membrane affinity have tremendous potential to serve as long-acting pain-killers.

## Results

### Single-knots wash-off faster and possess a much lower affinity for native membranes when compared to wild-type DkTx

**Figure 2:**
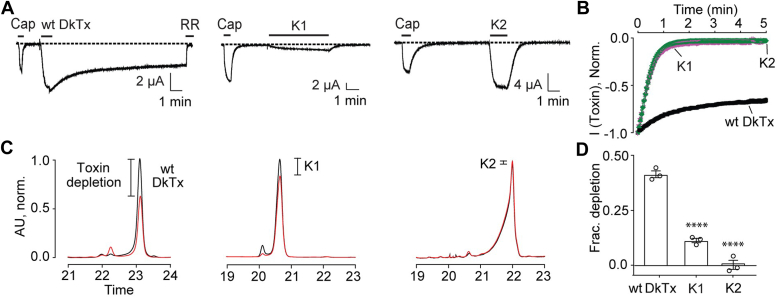
**Functional and membrane affinity characterization of wild-type DkTx and its individual knots.***A*, representative electrophysiological recordings depicting TRPV1 currents obtained upon the application of the small molecule TRPV1-agonist, capsaicin (5 μM), followed by wild-type DkTx (20 μM, *left*), K1 knot (20 μM, *center*), and K2 knot (20 μM, *right*) to *Xenopus laevis* oocytes injected with rat TRPV1 mRNA. Wash-off was performed by starting buffer perfusion after the currents saturated in presence of the agonists. In the case of wild-type DkTx, the TRPV1 blocker, ruthenium red (RR, 7 μM), was applied to completely inhibit TRPV1 currents at the end of the recording. *B*, averaged normalized wash-off current traces obtained with 20 μM wild-type DkTx (*black*), K1 (*purple*), and K2 (*green*); n = 3 with SEM depicted in each case. *C*, representative reverse-phase HPLC chromatograms depicting toxin depletion upon incubation of wild-type DkTx (*left*), K1 knot (*center*), and K2 knot (*right*) with uninjected *Xenopus laevis* oocytes. Traces in *black* correspond to the controls wherein the toxins solubilized in a buffer devoid of oocytes were subjected to reverse-phase HPLC analyses and those in *red* were obtained from toxin solutions incubated with 100 uninjected oocytes. *D*, bar-scatter plot with SEM depicting a comparison of fractional depletion of wild-type DkTx (n = 3), K1 knot (n = 3), and K2 knot (n = 3) into uninjected *Xenopus laevis* oocytes. Individual knots were compared with wild-type DkTx for statistical analysis. ∗∗∗∗*p* ≤ 0.0001 (ANOVA followed by a multiple comparison test). “Wild-type” has been abbreviated as “wt”.

Consistent with previous reports (10, 17, 21), our electrophysiology experiments demonstrate that the single-knots wash off much faster than DkTx, with K2 possessing much higher potency for TRPV1 activation than K1 (Fig. 2*A*). Indeed, while the treatment of *Xenopus laevis* oocytes expressing rat TRPV1 with 20 μM of the K1 and K2 single-knots followed by buffer perfusion resulted in 97% and 99% attenuation of TRPV1 currents after 3 min of perfusion, respectively, a similar treatment with DkTx resulted in only 24% decrease in TRPV1 currents (Fig. 2*B* and Table 1).

**Table 1:** TRPV1 activation and membrane affinity data summary for all toxin variants

S. No.	Toxin	Expected mass (a.m.u)	Observed mass (a.m.u) (MALDI-TOF)	HPLC retention time (min)	EC_50_ (μM)	Fold change in EC_50_	Hill coefficient (n_H_)	% wash-off after 3 min of buffer perfusion post TRPV1 activation by 20 μM of toxin (unless otherwise stated)	Mol. Fraction partition coefficient (K_x_) in POPC/POPG vesicles	Fractional depletion upon incubation of 4 nmol toxin in 400 μl buffer containing 100 uninjected oocytes (unless otherwise stated)
1	wild-type DkTx	8586.0	8581.4	23.1	0.05 ± 0.01	1	1.4 ± 0.4	24.1 ± 3.2 45.3 ± 1.3 (3.3 μM toxin)	(8.2 ± 0.8)E6	0.42 ± 0.03 0.21 ± 0.05^a^
2	PYVPVT (Δ1)	8484.9	8482.9	23.2	0.67 ± 0.09	13	1.7 ± 0.4	21.2 ± 4.6	(6.0 ± 0.9)E6	0.42 ± 0.02
3	PYVPV (Δ2)	8383.8	8386.1	23.1	1.08 ± 0.14	22	1.5 ± 0.2	45.9 ± 5.5	(9.7 ± 1.3)E6	0.23 ± 0.02
4	PYVP (Δ3)	8284.6	8285.8	23.3	10.85 ± 2.20	217	1.6 ± 0.2	75.9 ± 6.2	(3.3 ± 0.3)E6	0.22 ± 0.03
5	PYV (Δ4)	8187.5	8188.9	23.3	8.64 ± 2.75	173	1.1 ± 0.1	67.7 ± 3.8	(1.9 ± 0.8)E6	0.17 ± 0.07
6	K1	4170.9	4171.9	20.2	C.N.D.	C.N.D.	C.N.D.	96.5 ± 1.9	(3.9 ± 1.1)E5^b^	0.11 ± 0.01
7	K2	3732.3	3735.8	21.9	3.33 ± 0.41	67	2.5 ± 0.9	98.6 ± 1.4	(2.0 ± 0.3)E4^b^	0.00 ± 0.03
8	SGTx	3834.4	3839.1	20.3	N.A.	N.A.	N.A.	N.A.	N.D.	N.D.
9	K1-SGTx	8687.1	8684.3	21.9	C.N.D.	C.N.D.	C.N.D.	67.3 ± 3.8	(3.7 ± 1.1)E7	0.28 ± 0.02
10	SGTx-K2	8248.5	8244.5	22.6	C.N.D.	C.N.D.	C.N.D.	70.2 ± 5.4	(4.8 ± 2.0)E7	0.22 ± 0.02
11	G_3_-DkTx	8700.1	8699.7	23.3	N.D.	N.D.	N.D.	N.D.	N.D.	N.D.
12	G_3_-(SGTx)_2_	8463.7	8469.2	22.1	N.A.	N.A.	N.A.	N.A.	N.D.	N.D.
13	DkTx-G_3_SLPETG_2_H_6_	10,221.7	10,238.12	22.5	N.D.	N.D.	N.D.	N.D.	N.D.	N.D.
14	(DkTx)_2_	17,989.7 (Na^+^ adduct)	17,995.7	24.7	0.25 ± 0.03	5	1.4 ± 0.2	5.2 ± 1.1 (3.3 μM toxin)	N.D.	0.45 ± 0.04^a^
15	DkTx-(SGTx)_2_	17,787.5	17,784.4	23.5	1.05 ± 0.12	21	0.8 ± 0.1	20.4 ± 1.0 (3.3 μM toxin)	N.D.	0.42 ± 0.04^a^

a fractional depletion upon incubation of 1 nmol toxin variants in 400 μl buffer containing 50 uninjected oocytes.

b K_x_ values reported by Swartz and coworkers (17), C.N.D.: could not be determined due to poor potency (only partial channel activation measurable at concentrations close to toxin solubility limit), N.A.: not applicable, N.D.: not determined.

Previously reported membrane affinity studies (discussed above) that yielded much lower molar partitioning (K_x_) values for the K1 and K2 single knots as compared to DkTx (17) were performed on artificial POPC/POPG vesicles that are quite distinct from physiological plasma membranes. Although the membrane partitioning trends obtained for DkTx and its variants from similar experiments have been demonstrated to faithfully recapitulate those obtained from the physiologically relevant membrane depletion experiments performed on *X. laevis* oocytes (18), we deemed it important to verify that this trend also holds for the single-knots. Subjecting the single-knots K1 and K2, and wild-type DkTx to toxin depletion assays on uninjected oocytes yielded fractional depletion values of 0.11, 0, and 0.42, respectively (Fig. 2, *C* and *D*), establishing that the single-knots possess a much lower affinity for native membranes as compared to DkTx, with the K1 knot possessing a higher membrane affinity as compared to the K2 knot, consistent with the membrane partitioning data (17).

### DkTx variants with truncated linkers wash off faster than wild-type DkTx but slower than the single-knots

**Figure 3:**
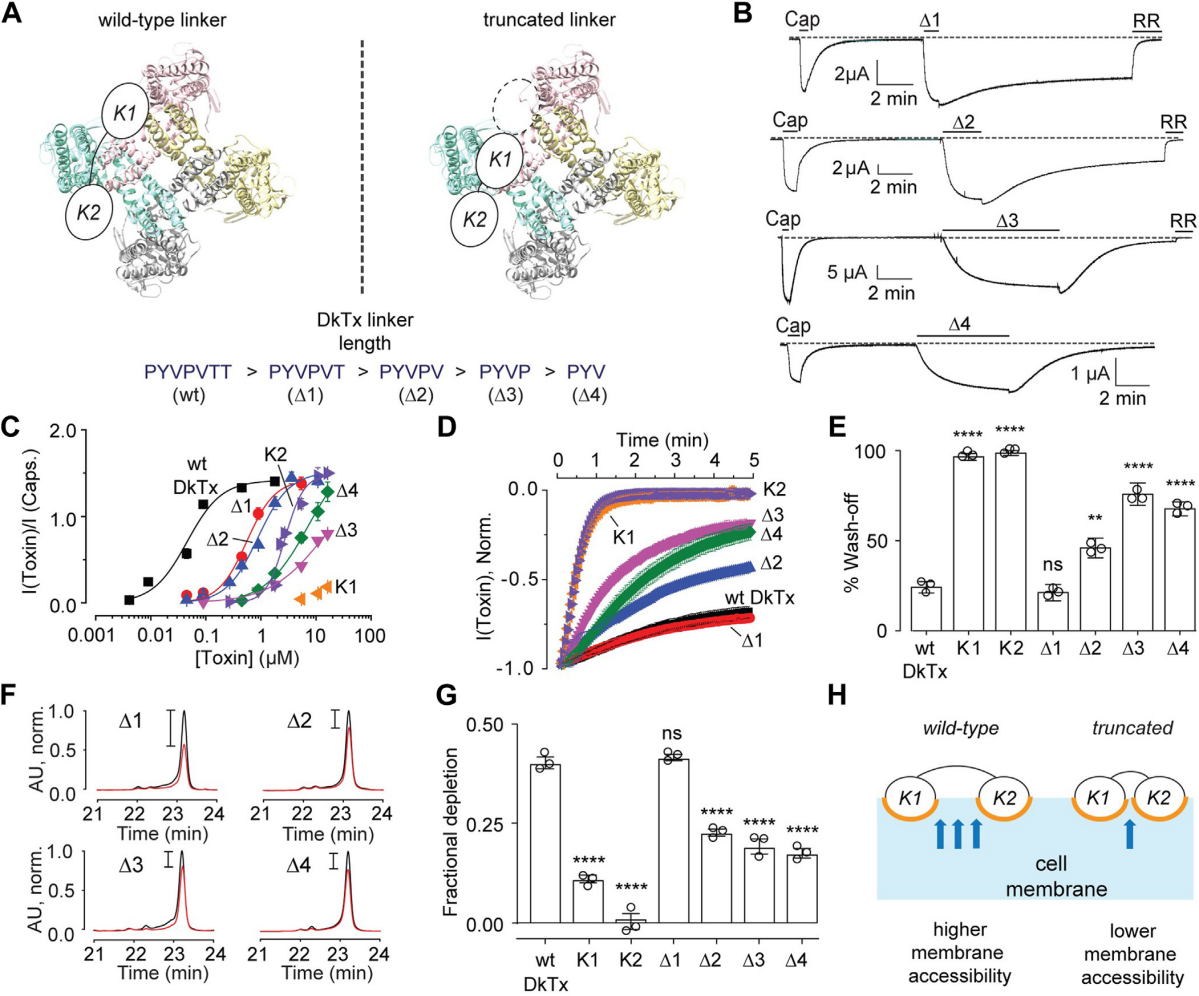
**Linker truncation variants of DkTx.***A*, *top*: Schematic representation of the experimental design for generating truncated linker-containing DkTx variants that are unable to bind bivalently to the channel. *Bottom*: DkTx truncation variants reported in this work. *B*, representative current traces for TRPV1 activation by the truncation variants (20 μM) followed by toxin wash-off by buffer perfusion. In the case of Δ1, Δ2, and Δ3, the TRPV1 blocker, ruthenium red (RR, 7 μM), was applied to completely inhibit TRPV1 currents at the end of the recording. *C*, dose–response plots for the truncation variants, wild-type DkTx, and the single-knots. Each data point corresponds to n ≥ 3 with SEM. *D*, averaged normalized wash-off current traces (n = 3 with SEM) obtained with 20 μM toxin concentrations. *E*, bar-scatter plot with SEM depicting the percentage reduction in current after 3 min of buffer perfusion post-toxin-mediated channel activation (n = 3 for each toxin). Individual knots and truncation variants were compared with wild-type DkTx for statistical analysis; ns: not statistically significant, ∗∗∗∗*p* ≤ 0.0001, ∗∗*p* ≤ 0.01 (ANOVA followed by a multiple comparison test). *F*, representative HPLC chromatograms depicting toxin depletion upon incubation of linker truncation variants (10 μM) with 100 uninjected *Xenopus laevis* oocytes (traces in *red* represent toxin solutions incubated with oocytes, and those in *black* correspond to the controls). *G*, bar-scatter plot with SEM depicting fractional depletion for wild-type DkTx (n = 3), the linker variants (n = 3), and the single knots (n = 3) obtained from the oocyte depletion experiments. Individual knots and truncation variants were compared with wild-type DkTx for statistical analysis. ns, not statistically significant, ∗∗∗∗*p* ≤ 0.0001 (ANOVA followed by a multiple comparison test). *H*, a schematic depiction of our reduced membrane accessibility hypothesis.

To study the contributions of membrane affinity and bivalency of DkTx in TRPV1 activation, we sought to disrupt the toxin’s bivalency while retaining its membrane affinity. We reasoned that one way of achieving this goal is to truncate the linker of DkTx that connects its two knots thereby preventing the concomitant binding of the knots of DkTx to their channel-binding sites (Fig. 3*A*, top). Such truncated linker-containing DkTx variants would be expected to possess wild-type DkTx-like membrane affinities as they retain the membrane-interacting portions of the toxin (the K1 and K2 knots). Alterations in the linker are not expected to hamper the toxin’s membrane affinity as both structural and functional data suggest that the linker does not participate in membrane interactions. Indeed, the DkTx-TRPV1 complex structure depicts the linker to be far removed from the membrane, in contrast to the K1 and K2 knots of the toxin that interact intimately with membrane lipids (16, 17). Moreover, drastically altering the properties of the linker by substituting its native sequence (PYVPVTT) with seven glycine residues results in minimal changes in the toxin’s membrane partitioning propensity (17), supporting the hypothesis that the linker does not directly contribute to membrane binding.

We generated DkTx linker truncation constructs wherein one through four *C*-terminal amino acids of the linker were sequentially deleted (Fig. 3*A*, bottom). All of these variants were TRPV1 agonists (representative electrophysiology traces are depicted in Fig. 3*B*), and dose–response analyses revealed that their potency decreased with shortening linker lengths, with the Δ3 and Δ4 linker variants demonstrating ∼200-fold lower potency as compared to wild-type DkTx (Fig. 3*C* and Table 1).

While the Δ1 variant exhibited similar wash-off percentages as compared to the wild-type toxin, the Δ2, Δ3, and Δ4 variants washed off faster (Fig. 3, *B*, *D* and *E*). Indeed, the Δ1 and the Δ2 variants demonstrated 21% and 46% TRPV1-current attenuation respectively after 3 min of buffer perfusion in comparison to the Δ3 and the Δ4 linker variants that demonstrated a 76% and 68% reduction of currents respectively over the same wash-off time period (Fig. 3*E*). This enhancement of the wash-off rates with the shortening of the linker implicates bivalency in contributing to the slow wash-off kinetics of DkTx, as the propensity for bivalent binding will progressively reduce as the linker is shortened. Notably, however, all of the truncation variants demonstrated lower wash-off percentages when compared to the single K1 and K2 knots (Fig. 3, *D* and *E*) suggesting that parameters other than valence also contribute to slow toxin wash-off. Indeed, if bivalency alone was responsible for slow wash-off, the monovalent Δ3 and Δ4 double-knots would be expected to wash off as fast as the monovalent single-knots. Interestingly, the membrane affinities of all of the truncation variants are higher than those of the single-knots (oocyte depletion studies depicted in Fig. 3*G*, and POPC/POPG vesicle experiments depicted in Fig. S4), supporting the hypothesis that membrane affinity is one of the parameters responsible for conferring slower wash-off kinetics to the truncated double-knots as compared to the single-knots.

In contrast to our expectations alluded to at the beginning of this section, the membrane affinities of the truncation variants are not identical to that of wild-type DkTx. Indeed, although the Δ1 variant depletes as proficiently into uninjected oocytes as wild-type DkTx, the Δ2, Δ3, and Δ4 variants deplete almost half as proficiently (Fig. 3*G*). These results suggest that in addition to conferring bivalency to DkTx, the linker also contributes to the toxin’s membrane affinity, albeit without directly interacting with the membrane. To explain this observation, we propose that the linker of wild-type DkTx allows the two knots to be spaced out far enough from each other such that efficient membrane solvation of both knots is achieved. Truncating the linker results in enhanced mutual proximity of the knots, limiting the toxin surface area accessible for membrane solvation leading to attenuation of the membrane affinity of these variants (Fig. 3*H*).

### Production of high membrane-affinity monovalent chimeric double-knot toxins

**Figure 4:**
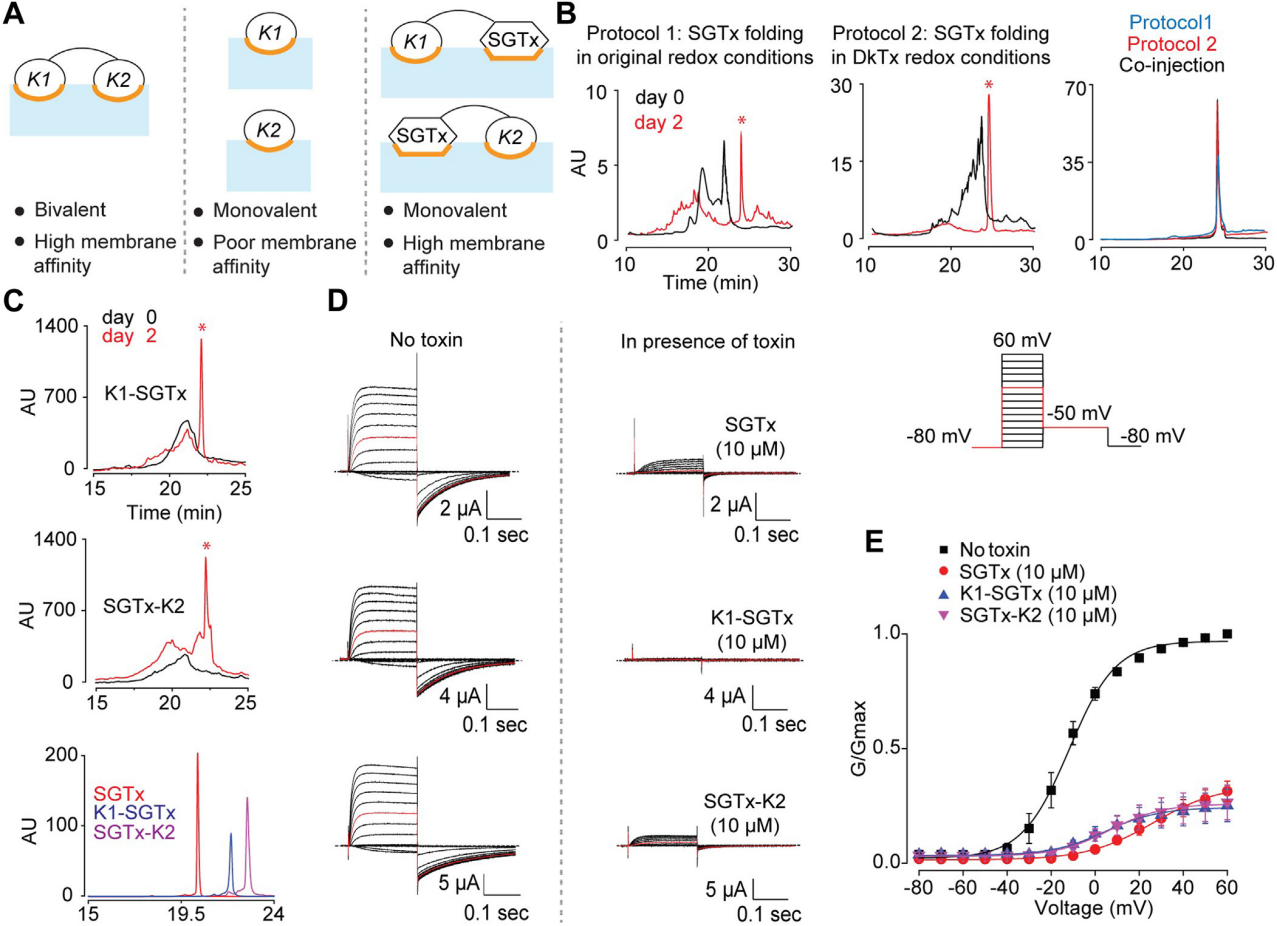
**Production and Kv2.1-inhibitory properties of the chimeric double-knot toxins.***A*, the TRPV1-binding valence and expected membrane interaction properties of wild-type DkTx, its single-knots, and the chimeric double-knots. *B*, HPLC chromatographic analyses of the time course of the folding of linear SGTx under the originally reported folding conditions (*left*), under DkTx-folding conditions (*middle*), and overlay of HPLC chromatograms obtained upon re-injection of the emergent peaks collected from both the approaches, and that obtained upon injection of a mixture of these peaks (co-injection). The emergent peaks are marked with *asterisks*. *C*, HPLC chromatograms for monitoring the folding of linear K1-SGTx (*top*) and linear SGTx-K2 (*middle*), and overlay of the chromatograms for purified folded SGTx, K1-SGTx, and SGTx-K2 (*bottom*). *D*, characterization of the K_v_2.1-modulatory activity of the chimeric knots. Oocytes injected with the mRNA of the agitoxin2-sensitive Kv2.1Δ7 channel were held at −80 mV and subjected to depolarizations ranging from −80 mV to 60 mV in 10 mV-increments, and the voltage was then stepped down to −50 mV to elicit tail currents (voltage protocol depicted in the *top right panel*). Agitoxin2-subtracted current traces before and after the application of the toxins (10 μM) are depicted, with the trace for the 10 mV-depolarization sweep in *red*. *E*, conductance (G)-Voltage (V) relationship plots obtained from the Kv2.1Δ7 recordings. Each data point corresponds to the averaged value of three experiments with SEM.

In addition to the linker-based approach described above, we employed another strategy aimed at disrupting the bivalency of the toxin while retaining its membrane affinity. This approach involved the generation of chimeric toxins by the replacement of each knot of DkTx one at a time with the Kv2.1 channel-inhibitor ICK toxin, SGTx (15, 22, 23) to form the K1-SGTx and SGTx-K2 double-knots, thereby precluding bivalent binding to TRPV1 (Fig. 4*A*). Our choice of SGTx was motivated by the observation that it partitions favorably into POPC/POPG vesicles (24), rendering it an ideal candidate for the creation of our desired monovalent yet high membrane affinity-possessing toxin variants.

To produce the desired K1-SGTx and SGTx-K2 chimeric toxins, it was imperative to identify a strategy that would yield properly folded versions of SGTx as well as of the K1 and K2 knots of DkTx. A comparison of conditions used previously for folding SGTx with those for DkTx revealed that although both employed identical ratios of oxidized and reduced glutathione, whereas folding of SGTx was performed in buffers containing ammonium acetate and urea (22), optimal folding of DkTx entailed the use of buffers containing detergents such as Triton X-100 (21). To evaluate whether SGTx will fold efficiently in DkTx-folding conditions, we produced linear SGTx using our standard procedure for generating linear DkTx (expressing the toxin in *E. coli* as a ketosteroid isomerase-N-G-toxin fusion protein followed by hydroxylamine treatment (18, 21)) and subjected it to DkTx-folding conditions. In parallel, we subjected linear SGTx to its standard folding conditions reported previously (22) and analyzed folding in both these cases by performing HPLC analyses. A sharp peak at the retention time of 24 min was observed in both the folding mixtures after 2 days (marked with an asterisk in Fig. 4*B*, left and middle panels). A co-injection experiment yielded a single prominent peak at the same retention time as those for the two peaks injected individually (Fig. 4*B*, right panel), suggesting that both the folding conditions yielded the same species. Next, we characterized the Kv2.1 channel-inhibitory properties of SGTx produced *via* the DkTx-folding protocol by performing two-electrode voltage clamp recordings on *X. laevis* oocytes expressing Kv2.1 (Fig. 4*D*). The resulting conductance-voltage (G-V) plot (Fig. 4*E*) demonstrated a profound rightward shift (of 38.3 mV) upon toxin application (Table S2), as expected for SGTx (22, 25), demonstrating that SGTx produced recombinantly by employing the DkTx production protocol yields the properly folded, active toxin.

Encouraged by our success in producing active SGTx under DkTx-folding conditions, we produced linear KSI-N-G-K1-SGTx and KSI-N-G-SGTx-K2 fusion proteins in *E. coli* and subjected them to those conditions post hydroxylamine-treatment. As with SGTx, a sharp peak emerged from both the folding mixtures within 2 days (Fig. 4*C*). MS analyses of these peaks (Fig. S3) gave *m/z* values that were close to the expected values for both toxins (for K1-SGTx, obtained: 8684.3, expected: 8687.1; for SGTx-K2, obtained: 8244.5, expected: 8248.5) and the application of 10 μM of K1-SGTx and SGTx-K2 resulted in profound Kv2.1-inhibition (Fig. 4*D*) with the former causing a 16.6 mV rightward shift in the G-V plot, and the latter affecting a 18.5-mV rightward shift (Fig. 4*E* and Table S2). These experiments established that the SGTx knots of these chimeric toxins were properly folded.

### The chimeric double-knots modulate both Kv2.1 and TRPV1, and wash off slower than the single-knots post TRPV1 activation

**Figure 5:**
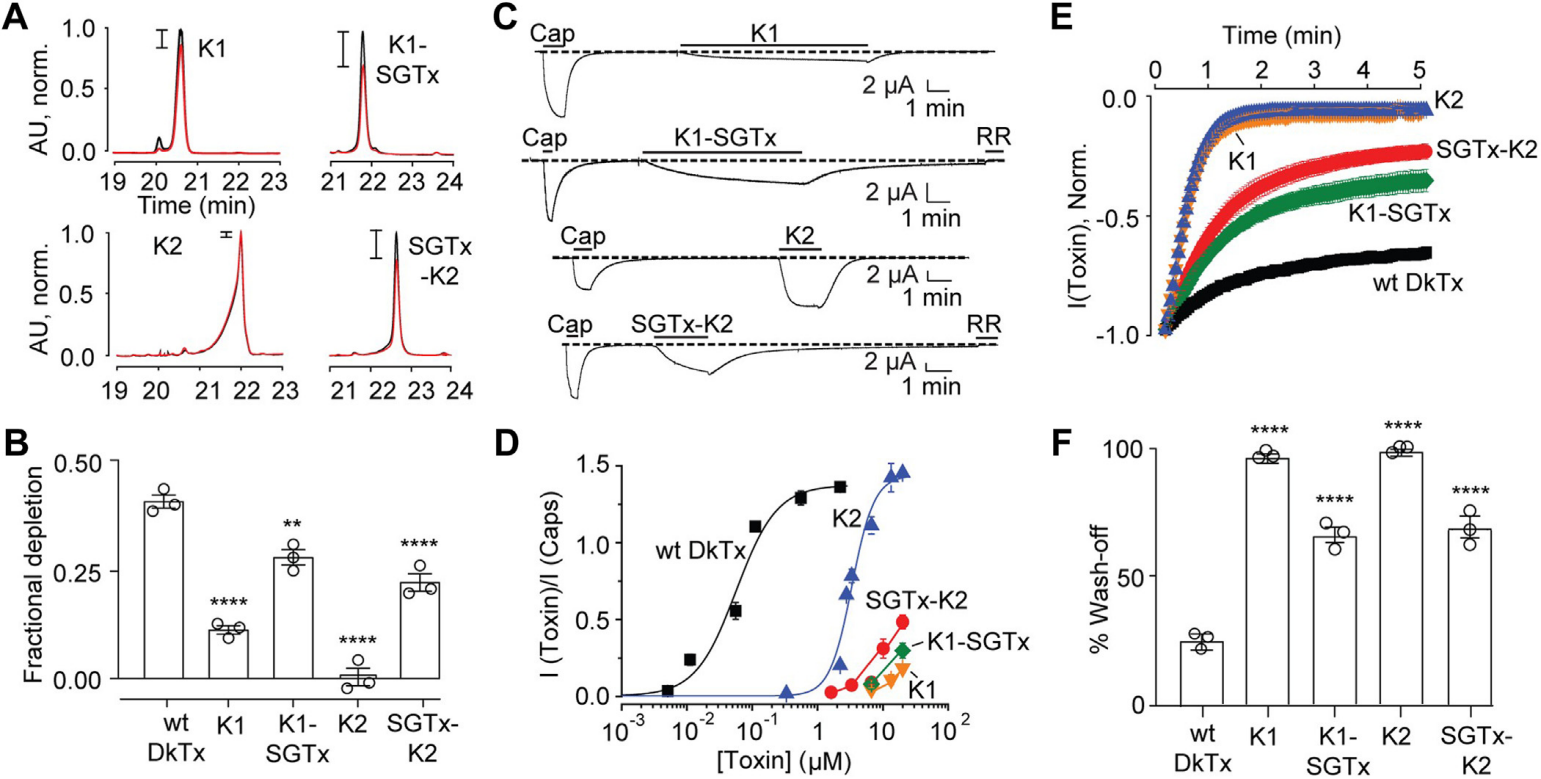
**Membrane affinity and TRPV1 activation characterization of chimeric double-knot toxins.***A*, representative HPLC traces depicting toxin depletion upon incubation of K1, K2, SGTx-K2, and K1-SGTx with uninjected *Xenopus laevis* oocytes. *B*, bar-scatter plot with SEM depicting fractional depletion for wild-type DkTx (n = 3), the chimeric double-knots (n = 3), and the single-knots (n = 3) obtained from the oocyte depletion experiments. Individual knots and chimeric double-knots were compared with wild-type DkTx for statistical analysis. ∗∗∗∗*p* ≤ 0.0001; ∗∗*p* ≤ 0.01 (ANOVA followed by a multiple comparison test). *C*, representative current traces for TRPV1 activation by the single knots and chimeric double-knot toxins (20 μM each) followed by toxin wash-off by buffer perfusion. In the case of chimeric double-knot toxins, the TRPV1 blocker, ruthenium red (RR, 7 μM) was applied to completely inhibit TRPV1 currents at the end of the recording. *D*, dose-response plots of the chimeric double-knots, wild-type DkTx, and the single-knots. Each data point corresponds to n ≥ 3 with SEM. *E*, averaged normalized wash-off current traces (20 μM toxin concentrations, n = 3 with SEM). *F*, bar-scatter plot with SEM depicting the percentage reduction in current after 3 min of buffer perfusion post-toxin-mediated channel activation (n = 3 for each toxin). Individual knots and chimeric double-knots were compared with wild-type DkTx for statistical analysis; ∗∗∗∗*p* ≤ 0.0001 (ANOVA followed by a multiple comparison test).

Both membrane partitioning and oocyte depletion studies revealed that as envisaged, K1-SGTx and SGTx-K2 possess much higher membrane affinity as compared to the K1 and K2 single knots, respectively. Indeed, the K_x_ values of these chimeric toxins were observed to be orders of magnitude higher than those of their respective single knots (Fig. S4 and Table 1), and they also yielded much higher fractional depletion values (0.22 for SGTx-K2 as compared to 0 for K2, and 0.28 for K1-SGTx as compared to 0.11 for K1; Fig. 5*B* and Table 1).

TRPV1 activation studies revealed that both K1-SGTx and SGTx-K2 could activate TRPV1 (Fig. 5, *C* and *D*) demonstrating that their K1 and K2 knots were folded properly. Notably, both K1-SGTx and SGTx-K2 washed off slower than the single-knots (Fig. 5*E*). Indeed, as opposed to the K1 and K2 single-knots that demonstrated complete attenuation of 20 μM toxin-evoked TRPV1 currents within 3 min of buffer perfusion, identical experiments with K1-SGTx and SGTx-K2 yielded only 67% and 70% attenuation of TRPV1 currents, respectively (Fig. 5*F*).

These results provide valuable insights into the roles of valence and membrane partitioning in the sustained mode of TRPV1 activation by DkTx. The observation that appending a membrane partitioning moiety (SGTx) to the single-knots of DkTx slows down their wash-off rates establishes the important role of the membrane in endowing slow wash-off kinetics to DkTx, and provides strong evidence that bivalency alone is not responsible for the slow wash-off of DkTx.

### The design and production of tetra-knot variants of the DkTx

**Figure 6:**
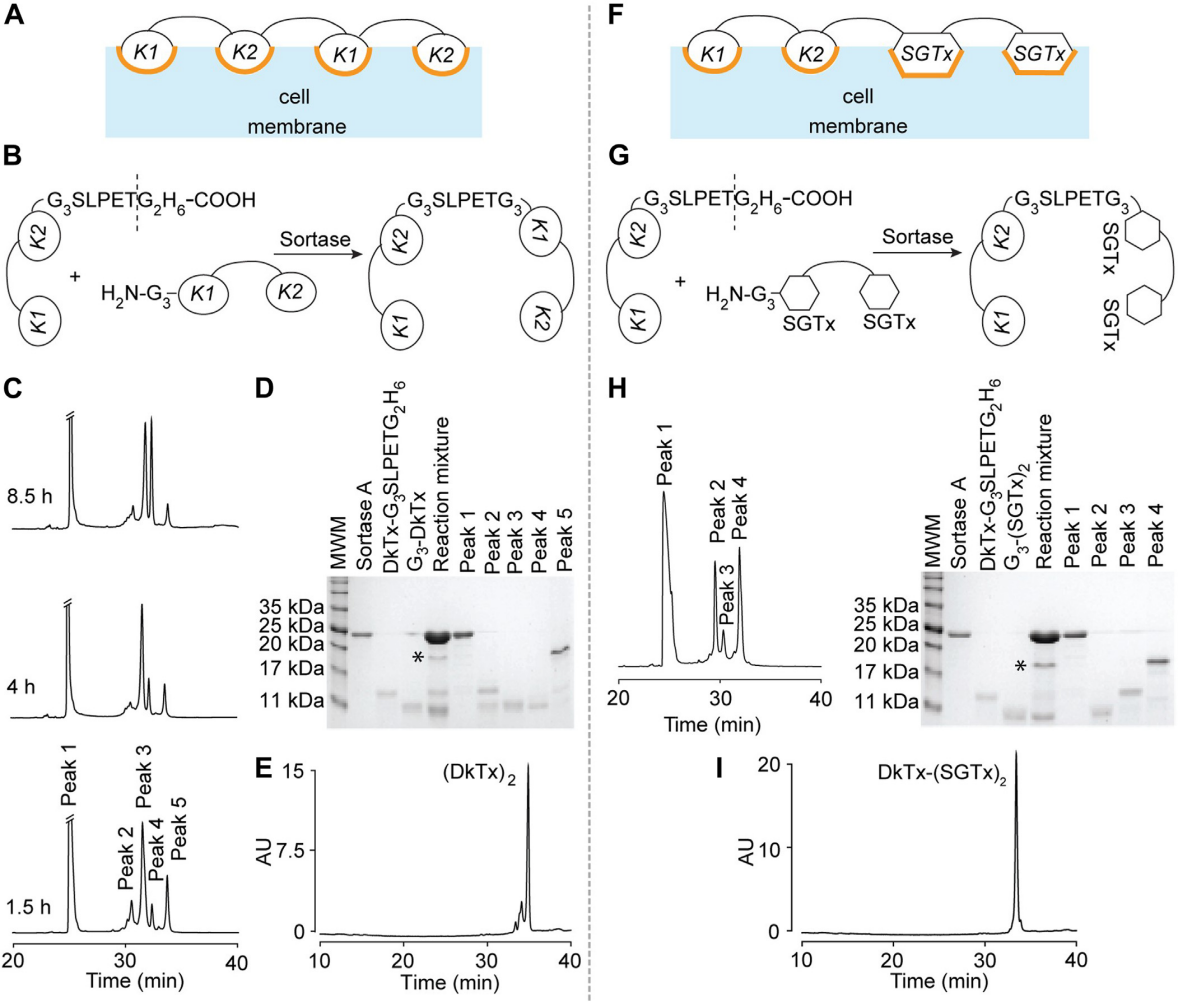
**Production of tetra-knot toxins.***A*, schematic representation of (DkTx)_2_ interacting with the cell membrane *via* its four membrane partitioning knots. *B*, schematic of the sortase A-mediated ligation reaction for producing (DkTx)_2_. *C*, optimization of the reaction time of sortase A ligation for the production of (DkTx)_2_. HPLC time course of the reaction between DkTx-G_3_SLPETG_2_H_6_ (25 μM) and G_3_-DkTx (37.5 μM) in the presence of sortase A (75 μM). *D*, SDS-PAGE analysis of all the collected peaks along with those for sortase A and the double-knot precursors. Peak 5 contained the desired product (DkTx)_2_ whereas peak 4 is for the hydrolysis side-product. *E*, purity analysis HPLC chromatogram for (DkTx)_2_. *F*, schematic representation of (DkTx)-(SGTx)_2_ interacting with the cell membrane. *G*, schematic of the sortase A-mediated bioconjugation reaction for producing DkTx-(SGTx)_2_. *H*, *left*: HPLC chromatogram for the reaction mixture containing DkTx-G_3_SLPETG_2_H_6_ (25 μM) and G_3_-(SGTx)_2_ (37.5 μM) in the presence of sortase A (75 μM) after 1.5 h of reaction time. *Right*: SDS-PAGE analysis of peaks collected from the HPLC chromatogram on the *left* showing a band for DkTx-(SGTx)_2_ at the expected position on the gel (peak 4), and those for the unreacted double-knots, and sortase A. *I*, purity analysis HPLC chromatogram for DkTx-(SGTx)_2_.

Considering the important role of the membrane affinity of DkTx in endowing it with slow wash-off rates, we reasoned that enhancing the membrane affinity of DkTx should slow down its wash-off. To test this hypothesis, we developed the tetra-knot toxins, (DkTx)_2_ and DkTx-(SGTx)_2_, possessing four membrane partitioning knots each, as opposed to two in DkTx (Fig. 6, *A* and *F*).

We envisioned that the presence of a large number of cysteines in our proposed tetra-knots (24 in each molecule) would render their production challenging due to the likelihood of these cysteines forming non-native mixed disulfides during folding. This apprehension was supported by our recent work on labeling DkTx with a fluorophore that revealed that introducing a non-native cysteine into DkTx results in the formation of misfolded toxin species (26). Therefore, instead of attempting to produce the tetra-knots by employing our standard protocol for producing DkTx that entails expressing the full-length linear protein in *E. coli* followed by subjecting it to redox folding conditions, we employed a step-wise approach involving the production of properly folded precursor double-knots followed by their enzymatic ligation to form the desired tetra-knots. We ligated the double-knots by using the sortase A enzyme that catalyzes the formation of a peptide linkage between proteins containing a *C*-terminal LPXTG motif (where X can be any amino acid) and those appended with an *N*-terminal G_3_ sequence (26, 27, 28, 29).

To produce (DkTx)_2_, we generated two DkTx variants: one appended with the G_3_SLPETG_2_H_6_ sequence on its *C*-terminus and the other containing the G_3_ sequence on its *N*-terminus and treated them with sortase A in order to form the desired tetra-knot containing the G_3_SLPETG_3_ linker between the two double-knots (Fig. 6*B*). HPLC chromatogram analyses of this mixture after 1.5 h, 4 h, and 8.5 h of reaction time yielded five peaks (Fig. 6*C*), and SDS-PAGE analysis of the mixture (Fig. 6*D*) gave a band near the expected molecular weight of the tetra-knot (∼18 kDa, marked with an asterisk). Subjecting the collected HPLC peaks to SDS-PAGE revealed that this species was eluting as peak 5 on the HPLC chromatogram (Fig. 6, *C* and *D*), and MALDI MS analysis of this peak gave an *m*/*z* value of 17,995.7 (Fig. S3), close to the expected molecular weight of the tetra-knot (17,989.8 Da). Peaks 2 and 3 of the HPLC chromatograms (Fig. 6*C*) corresponded to those for the unreacted double-knots, whereas sortase A eluted as peak 1. Peak 4 demonstrated a steady time-dependent increase in its intensity, coinciding with the reduction in the intensity of the peak for the desired tetra-knot (peak 5). MALDI MS characterization of this peak yielded an *m*/*z* value of 9280.3 which corresponds to that for DkTx-G_3_SLPET (expected mass: 9284.7 Da), the hydrolysis product of the thioester produced at the threonine of DkTx-G_3_SLPETG_2_H_6_ during the sortase reaction. The production of such a hydrolyzed side-product is a well-known limitation of sortase bioconjugation (27, 28, 29), and our reaction time course experiments (Fig. 6*C*) revealed that a reaction time of 1.5 h was optimal, yielding the highest yield for the desired tetra-knot (peak 5) and minimal yields for this undesired side product (peak 4). With these optimized conditions in hand, we proceeded to generate (DkTx)_2_ in high purity (HPLC trace for the purified toxin is depicted in Fig. 6*E*). We also produced DkTx-(SGTx)_2_ by ligating the DkTx-G_3_SLPETG_2_H_6_ and G_3_-SGTx-PYVPVTT-SGTx double-knots using the approach described above for (DkTx)_2_ (Fig. 6, *F*–*I*).

### The tetra-knots possess higher membrane affinity and slower wash-off rates than DkTx

**Figure 7:**
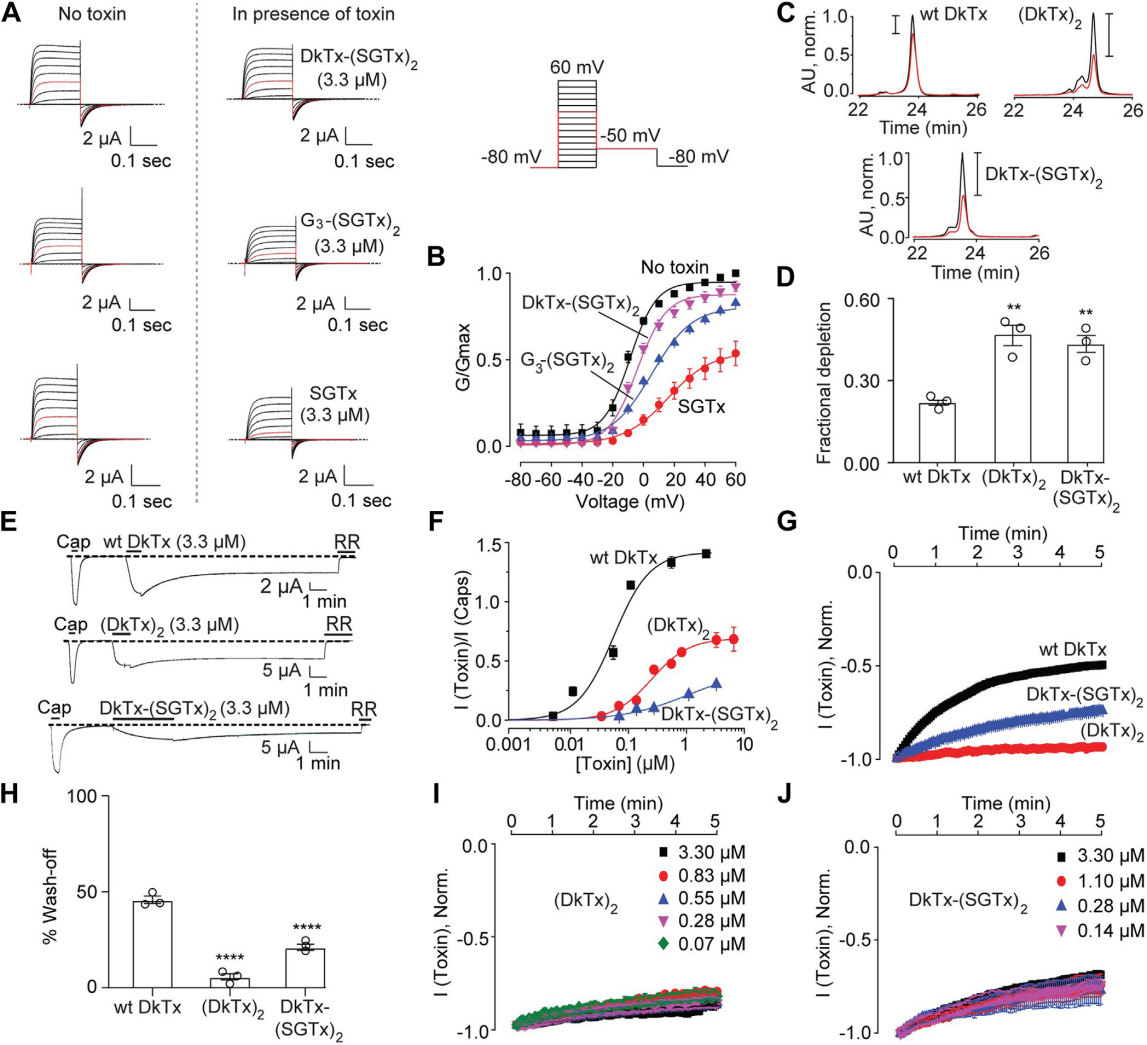
**Characterization of the tetra-knot toxins and their synthetic precursors.***A*, Kv2.1 channel-modulation by DkTx-(SGTx)_2_, G_3_-(SGTx)_2_, and SGTx. Oocytes expressing agitoxin2-sensitive Kv2.1Δ7 channel were held at −80 mV and subjected to depolarizations from −80 mV to 60 mV in 10 mV-increments and then hyperpolarized to −50 mV to elicit tail currents (voltage protocol on the *top right*). Agitoxin2-subtracted current traces before and after the toxin application (3.3 μM) are depicted, with the trace for the 10 mV-depolarization sweep in *red*. *B*, G-V relationships for Kv2.1Δ7 in the absence or presence of the toxins (3.3 μM; each data point: n = 3 with SEM). *C*, representative HPLC profiles, and (*D*) bar-scatter plot depicting fractional depletion for wild-type DkTx and the tetra-knots (n = 3 in each case) in uninjected *Xenopus laevis* oocytes. The tetra-knots were compared with wild-type DkTx for statistical analysis. ∗∗*p* ≤ 0.01 (ANOVA followed by a multiple comparison test). *E*, representative current traces for TRPV1 activation by wild-type DkTx, (DkTx)_2_ and DkTx-(SGTx)_2_ (3.3 μM each) followed by toxin wash-off by buffer perfusion. Ruthenium red (RR, 7 μM) was applied to completely inhibit TRPV1 currents. *F*, TRPV1 activation dose-response plots for wild-type DkTx, (DkTx)_2_ and DkTx-(SGTx)_2_. Each data point: n = 3 with SEM. *G*, averaged wash-off TRPV1 current traces (n = 3 with SEM) obtained with 3.3 μM toxin concentrations. *H*, bar-scatter plot with SEM depicting the percentage reduction in current after 3 min of buffer perfusion post toxin-mediated channel activation (n = 3). The tetra-knots were compared with wild-type DkTx for statistical analysis, ∗∗∗∗*p* ≤ 0.0001 (ANOVA followed by a multiple comparison test). *I*, wash-off studies on (DkTx)_2_ (n = 3 with SEM at each concentration). *J*, wash-off studies on DkTx-(SGTx)_2_ (n = 3 with SEM at each concentration).

To determine whether the SGTx knots in DkTx-(SGTx)_2_ and its precursor double-knot, G_3_-(SGTx)_2_, were properly folded and active, we characterized their Kv2.1-inhibitory properties using the voltage protocol depicted in Figure 7*A* (top right). We performed all studies with 3.3 μM or lower tetra-knot concentrations as DkTx-(SGTx)_2_ demonstrated limited solubility in the recording buffer at higher concentrations. The application of 3.3 μM of DkTx-(SGTx)_2_ resulted in Kv2.1 inhibition (the first panel from the top, Fig. 7*A*) shifting the Kv2.1 G-V plot rightward by 12.9 mV (Fig. 7*B* and Table S2). The same concentration of G_3_-(SGTx)_2_, also inhibited Kv2.1 (the second panel from the top, Fig. 7*A*), causing a rightward shift of 14.2 mV in the G-V plot of the channel (Fig. 7*B*). These Kv2.1-inhibitory effects of DkTx-(SGTx)_2_ and G_3_-(SGTx)_2_ established that their SGTx knots were properly folded.

Oocyte membrane depletion experiments revealed that as hypothesized, the tetra-knots possessed higher membrane affinities when compared to DkTx. Indeed, whereas incubating 50 uninjected oocytes in 2.5 μM of DkTx yielded a fractional depletion value of 0.21, (DkTx)_2_ and DkTx-(SGTx)_2_ depleted more than two-folds better, yielding fractional depletion values of 0.45 and 0.42 respectively (Fig. 7, *C* and *D*). The concentrations of the toxins used for these experiments were lower than those used for the depletion experiments on the single and double-knots described earlier that were performed with 10 μM concentrations of toxins due to the limited aqueous solubility of the tetra-knots.

TRPV1 activation studies on the tetra-knots revealed that both were TRPV1 agonists (Fig. 7, *E* and *F*) and possessed much lower potency for TRPV1 activation as compared to wild-type DkTx. Despite their lower potency as compared to wild-type DkTx, both the tetra-knots washed off substantially slower (Fig. 7*G*). Indeed, whereas wild-type DkTx (3.3 μM) demonstrated a 45% attenuation of TRPV1 currents after 3 min of buffer perfusion post-channel activation, identical experiments with (DkTx)_2_ and DkTx-(SGTx)_2_ yielded only 5% and 20% amelioration of TRPV1 currents respectively (Fig. 7*H*). Remarkably, wash-off experiments with a range of concentrations of the tetra-knots revealed that these toxins wash off extremely slowly at all the concentrations tested (Fig. 7, *I* and *J*) suggesting that they accumulate at high concentrations in the membrane even when applied at low concentrations, enabling the establishment of an efficient toxin relay during wash-off (Fig. 1*B*) rendering their wash-off rates concentration-independent.

These “gain-of-function” results obtained for the hyper-membrane affinity-possessing tetra-knots with respect to sustained TRPV1 activation convincingly establish the dominant role of the membrane affinity of DkTx in endowing it with this characteristic feature of its activity.

## Discussion

In this study, we have developed a suite of engineered DkTx-based toxin variants and characterized their TRPV1 activation and membrane affinity properties to elucidate the contributions of the toxin’s membrane affinity and valence towards endowing it with its sustained mode of TRPV1 activation. Our results unequivocally establish that the toxin’s high membrane affinity plays a central role in conferring it with its persistent TRPV1 activation property. Perhaps the strongest evidence in support of this conclusion is the slowing down of the wash-off rates of the single-knots of DkTx when appended to the Kv2.1 channel-targeting membrane partitioning toxin, SGTx (Fig. 5, *E* and *F*). These K1-SGTx and SGTx-K2 bifunctional chimeric double-knots inhibit Kv2.1 and activate TRPV1, and served as powerful tools for separating the effects of valence and membrane affinity as they were monovalent with respect to TRPV1, and yet possessed higher membrane affinities as compared to the single-knots. The importance of membrane affinity is reinforced by our hyper-membrane affinity-possessing tetra-knot variants, (DkTx)_2_ and DkTx-(SGTx)_2_, that wash off slower than wild-type DkTx (Fig. 7, *G* and *H*).

An unanticipated outcome of our experiments on the linker truncation variants of DkTx (Fig. 3) was the discovery that the linker contributes toward endowing high membrane affinity to the toxin without directly interacting with the membrane. The observation that truncating the linker results in attenuation of the toxin’s membrane affinity (Fig. 3*G*) supports the hypothesis that the enhanced proximity between the two knots of DkTx due to the shortening of the linker prevents membrane lipids from freely accessing the lipophilic regions of the knots of the toxin (Fig. 3*H*). Although DkTx variants containing truncated linkers have not been previously reported, two published studies have reported DkTx variants possessing non-native linkers. Swartz and co-workers produced a DkTx construct wherein the PYVPVTT native linker sequence was replaced with the flexible GGGGGGG sequence and used it to investigate the role of rigidity of the linker in its membrane partitioning propensity, reporting that this variant does not alter the toxin’s membrane affinity (17). More recently, Priel and co-workers replaced the seven amino acids-long linker of wild-type DkTx with an extended 14 amino acids-long linker and reported that increasing the linker length results in increased toxin-evoked TRPV1 channel conductance (30). Neither of these two studies, however, reported the impact of alterations to the DkTx linker on the toxin’s sustained channel-activation properties.

Although our results convincingly establish the role of the membrane in the sustained mode of TRPV1 activation demonstrated by DkTx, invoking the toxin’s high membrane affinity does not explain all our results. For example, K1 and K2 demonstrate similar wash-off rates (Fig. 2, *A* and *B*) despite the former possessing a higher membrane affinity as compared to the latter (Fig. 2, *C* and *D*). Another result that cannot be explained exclusively by the membrane affinity hypothesis is the observation that although both tetra-knots wash off slower than DkTx, (DkTx)_2_ washes off slower than DkTx-(SGTx)_2_ (Fig. 7, *G* and *H*) despite both demonstrating comparable membrane affinity values as measured by their depletion into uninjected oocytes (Fig. 7*D*). Considering that the affinity of the toxins for the channel will have an important role in how rapidly they unbind (wash-off), we believe that these observations can be explained by their differential binding affinities for TRPV1. Specifically, it is likely that K1 and DkTx-(SGTx)_2_ possess a substantially lower binding affinity for TRPV1 than K2 and (DkTx)_2_ respectively, explaining why K1 washes off as fast as K2 despite possessing a higher membrane affinity than K2, and DkTx-(SGTx)_2_ washes off faster than (DkTx)_2_ despite possessing a similar membrane affinity as (DkTx)_2_. This hypothesis is supported by the results of a previous study that had concluded that K1 possesses a lower affinity for TRPV1-binding as compared to K2, based on the results of experiments on the K1K1 and K2K2 homo double-knots (17). Moreover, (DkTx)_2_ would be expected to bind more tightly to TRPV1 when compared to DkTx-(SGTx)_2_ as it contains two TRPV1-binding units per molecule that can potentially simultaneously bind to a single TRPV1 homotetramer (as depicted in the structure of the DkTx-TRPV1 complex in Fig. 1*A*), as opposed to DkTx-(SGTx)_2_ that contains a single TRPV1-binding unit per molecule.

A notable aspect of our work is the development of unconventional engineered toxin variants such as the bifunctional chimeric double-knots and the tetra-knots. To the best of our knowledge, although artificial double-knot toxins containing two knots each targeting different domains of the same channel have been reported (29), those that can modulate the functions of two disparate channels (such as the K1-SGTx and SGTx-K2 that we describe herein), have not been previously reported. Additionally, (DkTx)_2_ and DkTx-(SGTx)_2_ constitute the first reported examples of toxins containing as many as four ICK motifs. Our successful use of sortase A-mediated ligation to produce these tetra-knots consolidates the steadily increasing number of reports that employ this approach to produce engineered versions of cysteine-rich toxins (18, 29). We believe that the protein engineering approaches that we have employed in this work can be effectively utilized to interrogate the roles of the membrane in the function of other membrane-interacting proteins, including other ion channel and receptor-targeting toxins. In particular, applying such approaches to toxins that demonstrate slow wash-off rates akin to DkTx, such as hanatoxin (31), GxTx-1E (32), and the recently discovered Nav channel agonist, excelsatoxin A (33), will enable evaluation of the roles of the membrane in the sustained mode of activity of these toxins as well. Taken together, these studies will address the intriguing hypothesis that endowing enhanced membrane affinity to membrane-interacting toxins is a general evolutionary strategy for achieving sustained modulation of channel and receptor proteins.

Apart from the insights on the mechanisms underlying TRPV1 activation, our results have potential ramifications for the development of TRPV1 agonists as analgesics. TRPV1 plays a central role in the pain-sensing pathway of the body, and the TRPV1 agonists, capsaicin and resiniferatoxin, are in clinical use for pain therapy (19, 20). While capsaicin is used in cream formulations for topical use, resiniferatoxin is administered intrathecally to treat severe pain in patients suffering from advanced stages of cancer. These TRPV1 agonists function as painkillers by first activating TRPV1 resulting in Ca^2+^ influx, leading to Ca^2+^-mediated channel desensitization (34), thereby attenuating pain. Considering the tremendous potential of TRPV1 agonists for use in pain therapy, it is tempting to speculate that the engineered highly persistent TRPV1 agonists such as the tetra-knot toxins we report in this study may serve as long-acting analgesics. It is important to note that the recordings reported herein were performed in Ca^2+^-free external solutions to prevent channel desensitization allowing us to observe the slow wash-off of the toxins. Under native conditions, however, the presence of Ca^2+^ would cause channel desensitization enabling these toxins to serve as analgesics. Nevertheless, one potential challenge for leveraging the sustained TRPV1 activation properties of these tetra-knots for pain therapy is their lower potency for channel activation as compared to DkTx (Fig. 7*F*). More generally, our observation that increasing the membrane affinity of DkTx results in even more persistent TRPV1 activation suggests that appropriately derivatizing other TRPV1 agonists including small molecules to endow them with enhanced membrane partitioning ability may lead to the development of long-acting analgesics.

## Experimental procedures

### The production of single and double-knot toxin variants

#### Mutagenesis and cloning

DkTx linker truncation variants were generated by whole-plasmid PCR mutagenesis on a plasmid containing the DkTx gene downstream of the ketosteroid isomerase (KSI) gene in a pET31b vector as previously described (18). SGTx, K1-SGTx, SGTx-K2, and SGTx-PYVPVTT-SGTx (referred to as (SGTx)_2_) DNA constructs cloned into the pUC57 vector were procured from GenScript Biotech, and were further subcloned downstream of the ketosteroid isomerase (KSI) gene in the pET31b vector, *via* restriction-free cloning.

#### Recombinant production of linear toxins

Toxin constructs were overexpressed in *E. coli* BL21(DE3) cells to produce linear proteins according to the workflow described previously (18). Briefly, the toxins were expressed as fusion proteins with ketosteroid isomerase (KSI) to direct their expression into bacterial inclusion bodies. The toxin and KSI protein sequences were separated by the N-G (Asn-Gly) linker enabling hydroxylamine-mediated cleavage at the N-G site to produce linear toxins appended with an *N*-terminal Gly (as depicted in Table S1) after pellet processing of the bacterial cells. The *E. coli* overexpression SDS-PAGE analyses for all single and double-knot variants are depicted in Fig. S1 of the supporting information section.

#### Folding of linear toxins and purification of folded toxins

Lyophilized linear single and double-knot toxins were folded under conditions optimized previously (18) that involved incubating them at 4 °C in redox refolding buffers containing Tris-HCl (0.4 M), EDTA (1 mM), Triton X-100 (0.5%), GSH (2.5 mM), and GSSG (0.25 mM) at pH 8.0 for 2 days. Folding of SGTx was attempted by employing this standard DkTx folding protocol, as well as by using a protocol that has been previously reported for SGTx (22) that involved the slow addition of a solution of the linear toxin (2 mg/ml dissolved in an aqueous solution of 50% acetonitrile containing 0.1% TFA) into a folding buffer at pH 7.8 containing ammonium acetate (0.1 M), urea (2 M), reduced glutathione (2.5 mM), and oxidized glutathione (0.25 mM), titrated by using NH_4_OH (7.7 N solution). The progress in folding was monitored by subjecting the folding mixtures to HPLC analyses on an Agilent 300SB-C18 RP-HPLC column (300 Å pore size) by employing a water-acetonitrile (0.1% TFA) linear gradient (5–65% acetonitrile over 30 min). The folded toxins were purified by HPLC, and the purity of the isolated folded toxins was also evaluated *via* HPLC. The folded toxins were characterized *via* MALDI-TOF. The HPLC traces for purity evaluations of all toxins, and their MALDI MS spectra are depicted in Figs. S2 and S3, respectively.

### The production of tetra-knots

His-tagged sortase A (Δ59) was produced as described previously (26). The (DkTx)_2_ and DkTx-(SGTx)_2_ tetra-knots were produced by performing sortase A-mediated ligation of precursor double-knot variants containing the LPETG motif on their *C*-terminus with those containing *N*-terminal poly-G sequences (Fig. 6, *B* and *G*). One of the precursor double-knots for making (DkTx)_2_ and DkTx-(SGTx)_2_ was the same, and contained the GGGSLPETGGHHHHHH (G_3_SLPETG_2_H_6_) sequence on the *C*-terminus of DkTx. The other precursor double-knot for (DkTx)_2_ contained the GGG (G_3_) sequence on the *N*-terminus of DkTx, whereas that for DkTx-(SGTx)_2_ was G_3_-SGTx-PYVPVTT-SGTx (abbreviated as G_3_-(SGTx)_2_). The (SGTx)_2_ construct cloned into the pUC57 vector was procured from GenScript Biotech and subcloned into a pET31b vector downstream of the KSI gene appended with G_3_ using restriction-free cloning. We produced the folded versions of these toxins by employing the same workflow as described above.

We produced (DkTx)_2_ *via* sortase A ligation reactions by dissolving DkTx-G_3_SLPETG_2_H_6_ (5 nmol) and G_3_-DkTx (7.5 nmol) in 100 μl of 2X reaction buffer containing Tris HCl (100 mM), NaCl (300 mM), and CaCl_2_ (20 mM) at pH 7.5. To this solution was added miliQ water (63 μl), and sortase A enzyme (37 μl of a 405.3 μM stock in a buffer containing Tris HCl (50 mM), NaCl (150 mM), MgCl_2_ (5 mM), and 10% (*v*/*v*) glycerol at pH 7.5), yielding a final sortase A concentration of 75 μM. The resultant mixture was incubated for 1.5 h/4 h/8.5 h at room temperature and then subjected to RP-HPLC on the 300SB C-18 column by employing the water-acetonitrile (0.1% TFA) mobile phase (acetonitrile percentage was increased linearly from 5% to 58.2% over 40 min). We used a similar procedure to produce DkTx-(SGTx)_2_ by ligating DkTx-G_3_SLPETG_2_H_6_ with G_3_-(SGTx)_2_.

### Sample preparation for MALDI-TOF MS characterization of toxins

MALDI-TOF mass spectrometry analyses were performed either on 4800 Plus MALDI TOF/TOF instrument from AB Sciex at the IISER Pune MALDI facility, or on a Bruker Nano-LC MALDI TOF/TOF spectrometer at the IISER Bhopal Central Instrumentation Facility. The matrix employed was either a mixture of 2,6-dihydroxyacetophenone (65.7 mM) and diammonium hydrogen citrate (56.9 mM) in 43% ethanol in water (35), or sinapic acid (44.6 mM) in dioxane.

### Two-electrode voltage clamp electrophysiology and data analysis

#### General

Oocytes were surgically removed from anesthetized adult female *X. laevis* in accordance with a protocol approved by the Institutional Animal Ethics Committee (IAEC), IISER Bhopal. Oocytes were then digested with collagenase type 2 enzyme (2 mg/ml) in calcium-free OR2 buffer containing NaCl (82.5 mM), KCl (2.5 mM), MgCl_2_ (1 mM) and HEPES (5 mM) at pH 7.6 for 1 h at 18 °C on an automated rocker and then de-folliculated with cut pipettes. The cRNA (rat TRPV1 or Kv2.1Δ7) was synthesized by *in vitro* transcription using HiScribe T7 ARCA mRNA Kit (New England Biolabs Inc). De-folliculated oocytes were injected with rTRPV1 or Kv2.1Δ7 cRNA solution (50 nl) in different dilutions (full strength to 1:4 dilution with RNase-free water) and stored at 18 °C in ND96 solution containing NaCl (96 mM), KCl (2 mM), HEPES (5 mM), MgCl_2_ (1 mM), CaCl_2_(1.8 mM) and gentamycin (50 μg/ml) at pH 7.6. Recordings were performed on oocytes 1 to 2 days post-RNA injection under voltage-clamp conditions by using a two-electrode voltage clamp instrument (Warner Instruments) in a 150 μl recording chamber. The microelectrode (filled with 3 M KCl) resistances were between 0.1 to 1.2 MΩ.

#### TRPV1 recordings

The recordings were performed *via* the gap-free protocol by holding the oocytes at −60 mV, employing extracellular solutions containing NaCl (50 mM), KCl (50 mM), MgCl_2_ (1 mM), BaCl_2_ (0.3 mM), and HEPES (20 mM) at pH 7.4. The data were filtered at 0.5 kHz and digitized at 2.5 kHz.

Toxin-induced currents were normalized against the currents obtained upon a prior application of 5 μM capsaicin to the same oocyte. Subsequently, these I_Toxin_/I_Caps_ values were plotted against the concentration of the toxin and the Hill equation (below) was fit to the data using Origin 9.0.

$${y=A}_{\max}\;\left\{\left(x^{n}\right)/\left(k^{n}{+x}^{n}\right)\right\}$$

where, A_max_ is the I_Toxin_/I_Caps_, k is EC_50_ and n is the Hill coefficient.

To generate the normalized toxin wash-off plots depicted in Figures 2*B*, 3*D*, 5*E*, 7*G*, *I* and *J*, the data for 5 min of buffer perfusion post toxin-mediated channel activation was procured from the TEVC recordings in the axon test file (.atf) format using the Clampfit 10.5 software. This data was subjected to data point extraction at every 7500th point interval using the R3.4.1 package, and the current at each time point was normalized to the peak current value of the same recording (at the buffer perfusion initiation time point). The normalized data obtained from 3 to 5 recordings were used to obtain averaged traces for wash-off plots and plotted to generate the figures. The SEM values at each time point were plotted as error bars.

#### Kv2.1Δ7 recordings

The recordings were performed by employing step-voltage protocols, and the data were filtered at 1 kHz and digitized at 10 kHz. The extracellular solutions for Kv2.1 recordings contained KCl (50 mM), NaCl (50 mM), MgCl_2_ (1 mM), CaCl_2_ (0.3 mM), and HEPES (20 mM) at pH 7.4. Capacitive and background currents were identified by first blocking the Kv2.1 channel with agitoxin2 (36), and then subtracting them to generate the currents shown in Figures 4*D* and 7*A*. Conductance (G)-Voltage (V) relationships depicted in Figures 4*E* and 7*B* were obtained by measuring tail currents following depolarization to test voltages followed by fitting a single Boltzmann function to the data according to the equation, G/G_max_ = [1 + exp(-zF(V-V_1/2_)/RT)]^−1^ where z is the valence, F is the Faraday constant, R is the gas constant, and T is the temperature.

### Membrane affinity experiments on large unilamellar vesicles (LUVs) and uninjected *X. laevis* oocytes

#### Tryptophan fluorescence-based experiments on LUVs for double knots

Tryptophan fluorescence-based experiments on LUVs composed of POPC:POPG (1:1) were performed based on the methodological workflow described earlier (18, 37, 38, 39).

#### Toxin depletion assay for single and double-knots

Toxin depletion experiments depicted in Figures 2*C*, 3*F*, and 5*A* were performed as reported previously (18). Specifically, uninjected defolliculated stage V or VI oocytes (100 nos.) were incubated with 4 nmol of toxin dissolved in a 400 μl solution of HEPES (20 mM) buffer at pH 7.4 containing NaCl (50 mM), KCl (50 mM), MgCl_2_ (1 mM), and BaCl_2_ (0.3 mM) for 1 h at room temperature. Control experiments involved incubating the toxin under identical conditions without oocytes. Subsequently, the supernatant (200 μl) was removed and spiked with a solution of 2-nitrophenol (2.5 μl of a 2.5 mg/ml solution in water) used as an internal standard. A 100 μl aliquot of this mixture was injected into the reverse phase HPLC column (Agilent 300SB-C18 column, 300 Å pore size) and eluted using a water-acetonitrile (0.1% TFA) linear gradient (5–65% acetonitrile over 30 min). This experimental protocol was performed three times and the fractional depletion was calculated as follows:

$$\text{Fraction}\;\text{depletion}=\left(R_{\text{control}}{-R}_{\text{oocytes}}\right)/R_{\text{control}}$$

R_control_ is the averaged ratio of the area under the peak for the toxin and the area under the peak for 2-nitrophenol in the control experiments, and R_oocytes_ is the ratio of the same parameters obtained from the oocyte samples (n = 3).

#### Toxin depletion assay for tetra-knots

Uninjected defolliculated stage V or VI oocytes (50 nos.) were incubated with (DkTx)_2_ or DkTx-(SGTx)_2_ (1 nmol) in a 400 μl solution of HEPES (20 mM) buffer at pH 7.4 containing NaCl (50 mM), KCl (50 mM), MgCl_2_ (1 mM) and BaCl_2_ (0.3 mM) for 1 h at room temperature. Control experiments involved incubating the toxin under identical conditions without oocytes. Subsequently, the supernatant (300 μl) was removed and spiked with a solution of 2-nitrophenol (1.25 μl of a 2.5 mg/ml solution in water) used as an internal standard. A 250 μl aliquot of this mixture was injected into the reverse-phase HPLC column (Agilent 300SB-C18 column, 300 Å pore size) and eluted using a water-acetonitrile (0.1% TFA) linear gradient (5–65% acetonitrile over 30 min). The fractional depletion was calculated by using the same equation as depicted above.

### Statistical analysis

All statistical data were calculated using Origin 9 software and Prism 9 (GraphPad Software). For electrophysiology data analysis (percentage wash-off data comparison), and fractional depletion comparison, ANOVA followed by a multiple comparison test was used to determine statistical significance. The asterisks indicate values: ∗∗∗∗*p* ≤ 0.0001, ∗∗∗*p* ≤ 0.001, ∗∗*p* ≤ 0.01, ∗*p* ≤ 0.1, and ns: not statistically significant.

## Data availability

All data are contained within the manuscript's main text and the supporting information section.

## Supporting information

This article contains supporting information.

## Conflict of interest

The authors declare that they have no conflicts of interest with the contents of this article.
