# Supplementary material for: Dissecting the contributions of membrane affinity and bivalency of the spider venom protein DkTx to its sustained mode of TRPV1 activation
Source: J Biol Chem. 2023 Jun 10;299(7):104903. doi: 10.1016/j.jbc.2023.104903 (PMC10404664; doi:10.1016/j.jbc.2023.104903)
Supplement: Supporting Figures S1–S4 and Tables S1 and S2 [file mmc1.pdf]

Supporting Information

Dissecting the contributions of membrane affinity and bivalency of the spider venom protein DkTx to its sustained mode of TRPV1 activation

Yashaswi Singh,[1,2] Debayan Sarkar,[1,2] Subhadeep Duari,[2] Shashaank G.,[2] Pawas Kumar Indra Guru,[2] Hrishikesh, M. V.,[2] Dheerendra Singh,[1] Sahil Bhardwaj,[2] Jeet Kalia[1,2,3,4*]

[1]Department of Biology, Indian Institute of Science Education and Research (IISER) Pune, Dr. Homi Bhabha Road, Pashan, Pune–411008, Maharashtra, India

[2]Department of Biological Sciences, Indian Institute of Science Education and Research (IISER) Bhopal, Bhopal Bypass Road, Bhauri, Bhopal–462066, Madhya Pradesh, India

[3]Department of Chemistry, Indian Institute of Science Education and Research (IISER) Pune, Dr. Homi Bhabha Road, Pashan, Pune–411008, Maharashtra, India

[4]Department of Chemistry, Indian Institute of Science Education and Research (IISER) Bhopal, Bhopal Bypass Road, Bhauri, Bhopal–462066, Madhya Pradesh, India

*Correspondence may be addressed to:

Dr. Jeet Kalia
E-mail: jeet@iiserb.ac.in
Phone: +91-755-269-1437

Table of contents

Figure/Table	Page No.
Figure S1: Induction gels for KSI (keto steroid isomerase)-toxin fusion protein overexpression	3
Figure S2: HPLC traces for purity evaluation of toxins	4
Figure S3: MALDI-TOF mass spectra of toxins	5
Figure S4: Membrane affinity characterization of DkTx variants by tryptophan fluorescence assays on LUVs	6
Table S1: Sequences of all the toxins produced in this study	7
Table S2: Summary of the Kv2.1Δ7 inhibitory activity of the toxins	8

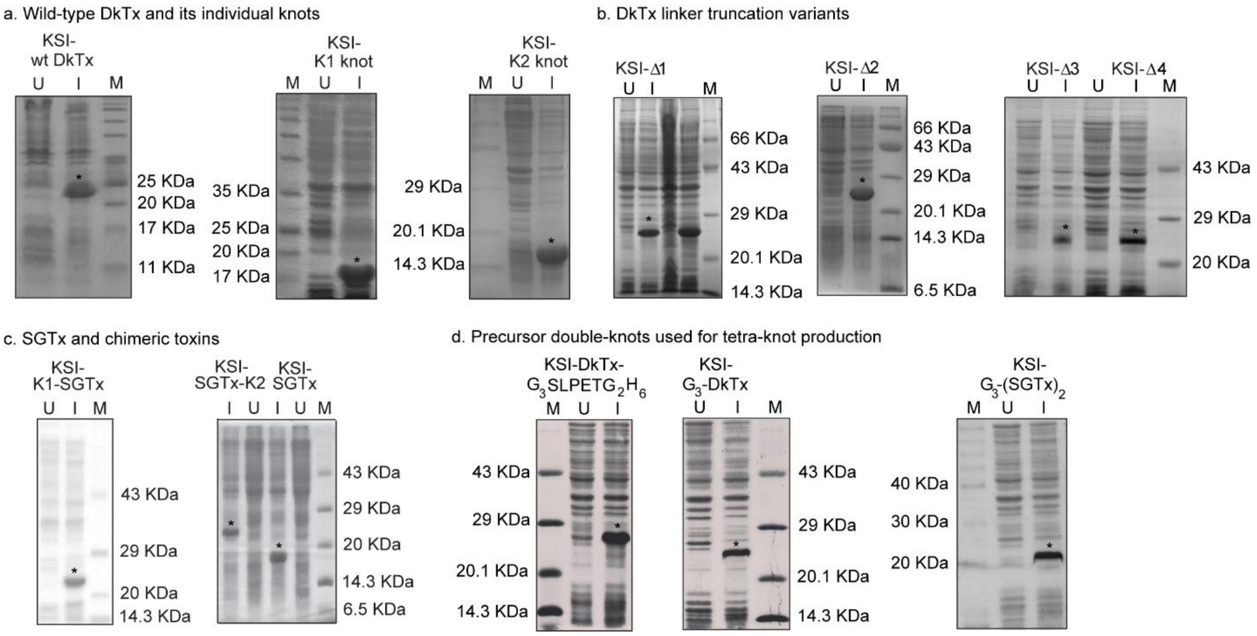

Figure S1: Induction gels for KSI (keto steroid isomerase)-toxin fusion protein overexpression. The bands for the overexpressed fusion proteins are marked with an asterisk on the gel image. (a) Wild-type DkTx and its individual knots: The expected molecular weight of KSI-wt DkTx is 22.6 KDa, KSI-K1 is 18.2 KDa, KSI-K2 is 17.8 KDa. (b) DkTx linker truncation variants: The expected molecular weight of KSI-Δ1 is 22.5 KDa, KSI-Δ2 is 22.4 KDa, KSI-Δ3 is 22.3 KDa, KSI-Δ4 is 22.2 KDa. (c) SGTx and chimeric toxin variants: The expected molecular weight of KSI-K1-SGTx is 22.7 KDa, KSI-SGTx-K2 is 22.3 KDa, and KSI-SGTx is 17.9 KDa. (d) Precursor double-knots used for tetra-knot production: The expected molecular weight of KSI-DkTx-$G_3SLPETG_2H_6$ is 24.3 KDa, KSI-G_3-DkTx is 22.8 KDa, and KSI-G_3-$(SGTx)_2$ is 22.5 KDa. U: uninduced cell lysate; I: induced cell lysate; M: molecular weight markers.

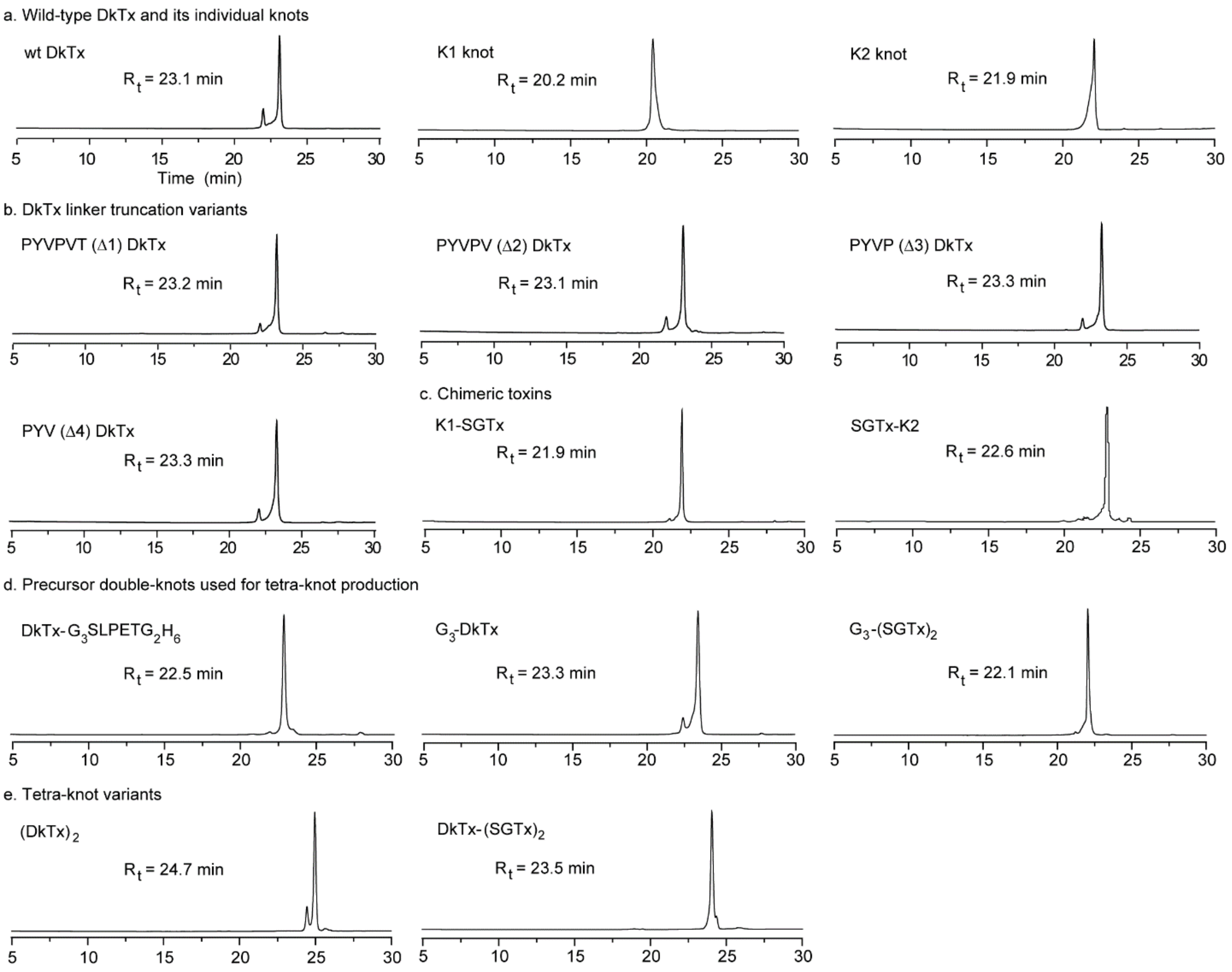

Figure S2: HPLC traces for purity evaluation of toxins. The retention times (R_t) are depicted along with each HPLC trace.

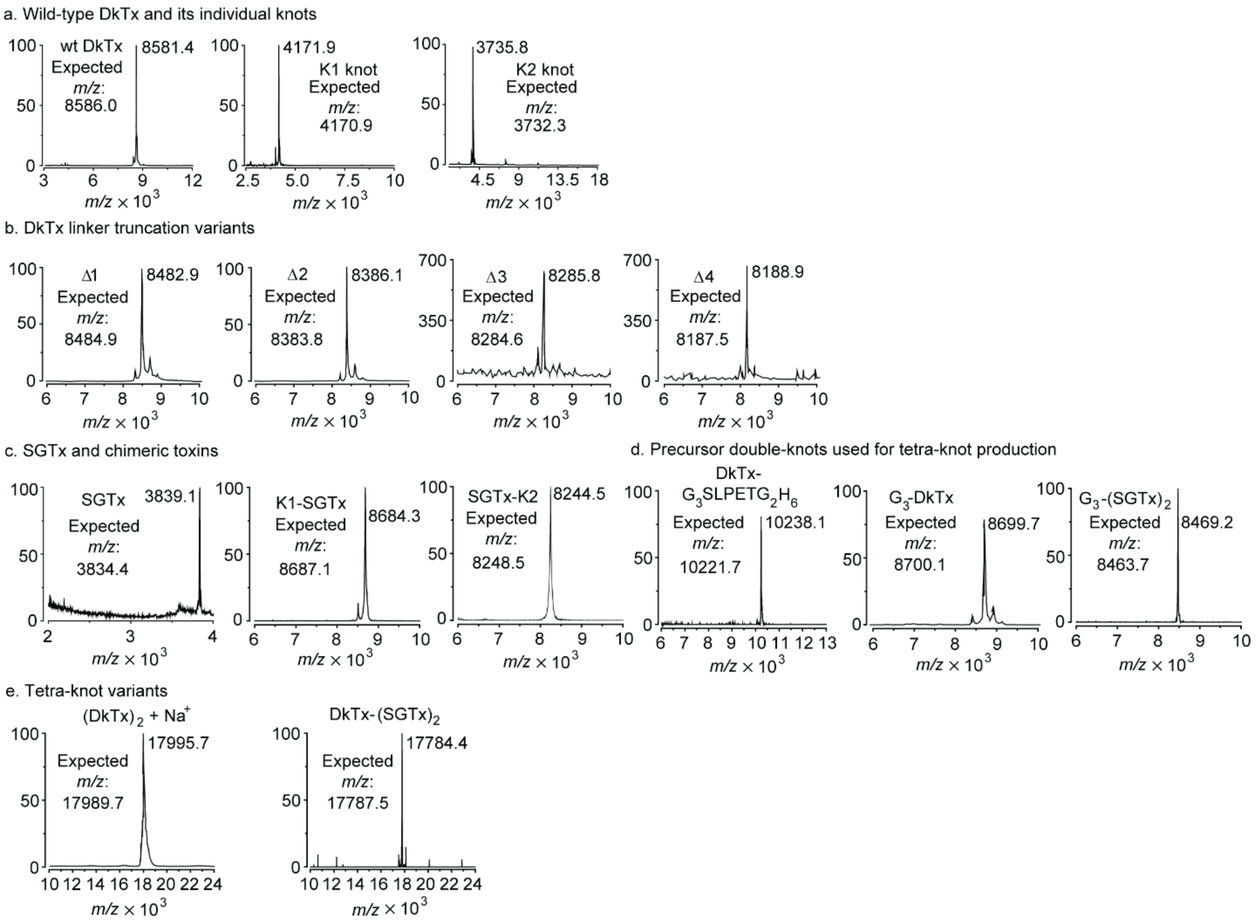

Figure S3: MALDI-TOF mass spectra of toxins. The expected and observed *m/z* values are provided in each panel.

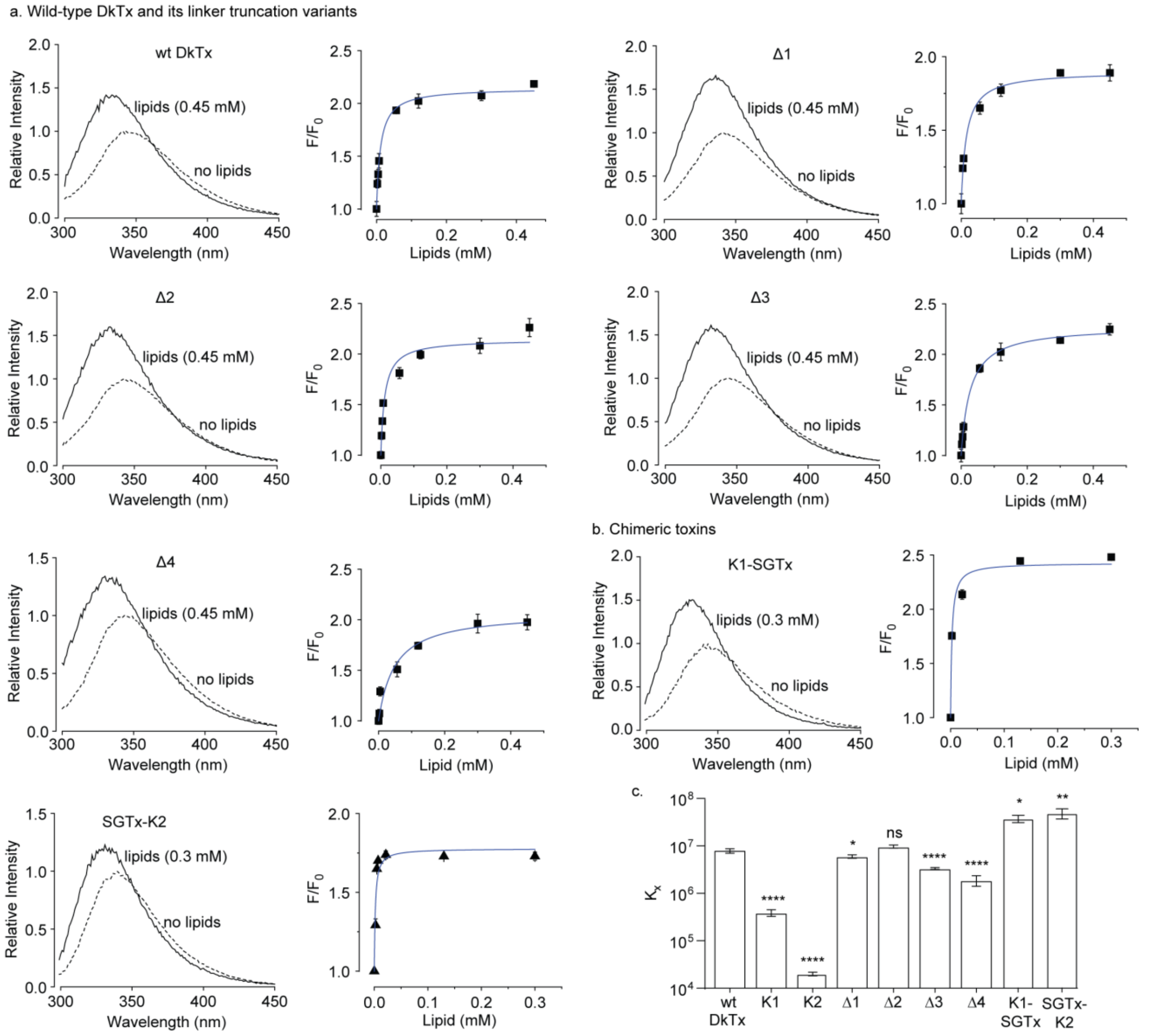

Figure S4: Membrane affinity characterization of DkTx variants by tryptophan fluorescence assay on LUVs. Tryptophan emission spectra of wt DkTx and its variants (left panels) in absence (dash lines) and in presence (solid lines) of LUVs made from a 1:1 molar ratio of POPC: POPG. All depicted solid traces for wt DkTx and its linker truncation variants (a) represent emission spectra in presence of 0.45 mM lipids, while that for the chimeric toxins (b) represent emission spectra at 0.3 mM lipids. Relative fluorescence intensity (right panels) at 320 nm (F/F_0) as a function of the available lipid concentration (60% of total lipid concentration). (c) Bar graph depicting K_x values obtained from these experiments. Individual knots, truncation variants and chimeric toxins were compared with wild-type DkTx for statistical analysis. ns: not statistically significant, ****P≤0.0001, **P≤0.01, *P≤0.1, ns: not significant (ANOVA followed by a multiple comparison test). K_x values for the individual knots (K1 and K2) are taken from Swartz and coworkers (main text reference number 17).

Table S1: Sequences of all the toxins produced in this study

Construct	Sequence
wild-type DkTx	GDCAKEGEVCSWGKKCCDLDNFYCPMEFIPHCKKYKPYVPVTTNCAKEGEVCGWGSKCCHGLDCPLAFIPYCEKYR
PYVPVT (Δ1) DkTx	GDCAKEGEVCSWGKKCCDLDNFYCPMEFIPHCKKYKPYVPVTNCAKEGEVCGWGSKCCHGLDCPLAFIPYCEKYR
PYVPV (Δ2) DkTx	GDCAKEGEVCSWGKKCCDLDNFYCPMEFIPHCKKYKPYVPVNCAKEGEVCGWGSKCCHGLDCPLAFIPYCEKYR
PYVP (Δ3) DkTx	GDCAKEGEVCSWGKKCCDLDNFYCPMEFIPHCKKYKPYVPNCAKEGEVCGWGSKCCHGLDCPLAFIPYCEKYR
PYV (Δ4) DkTx	GDCAKEGEVCSWGKKCCDLDNFYCPMEFIPHCKKYKPYVNCAKEGEVCGWGSKCCHGLDCPLAFIPYCEKYR
K1	GDCAKEGEVCSWGKKCCDLDNFYCPMEFIPHCKKYK
K2	GNCAKEGEVCGWGSKCCHGLDCPLAFIPYCEKYR
SGTx	GTCRYLFGGCKTTADCCKHLACRSDGKYCAWDGTF
K1-SGTx	GDCAKEGEVCSWGKKCCDLDNFYCPMEFIPHCKKYKPYVPVTTTCRYLFGGCKTTADCCKHLACRSDGKYCAWDGTF
SGTx-K2	GTCRYLFGGCKTTADCCKHLACRSDGKYCAWDGTFPYVPVTTNCAKEGEVCGWGSKCCHGLDCPLAFIPYCEKYR
G_3-DkTx	GGGDCAKEGEVCSWGKKCCDLDNFYCPMEFIPHCKKYKPYVPVTTNCAKEGEVCGWGSKCCHGLDCPLAFIPYCEKYR
G_3-$(SGTx)_2$	GGGTCRYLFGGCKTTADCCKHLACRSDGKYCAWDGTFPYVPVTTTCRYLFGGCKTTADCCKHLACRSDGKYCAWDGTF
DkTx-$G_3SLPETG_2H_6$	GDCAKEGEVCSWGKKCCDLDNFYCPMEFIPHCKKYKPYVPVTTNCAKEGEVCGWGSKCCHGLDCPLAFIPYCEKYRGGGSLP ETGGHHHHHH
$(DkTx)_2$	GDCAKEGEVCSWGKKCCDLDNFYCPMEFIPHCKKYKPYVPVTTNCAKEGEVCGWGSKCCHGLDCPLAFIPYCEKYRGGGSLP ETGGGDCAKEGEVCSWGKKCCDLDNFYCPMEFIPHCKKYKPYVPVTTNCAKEGEVCGWGSKCCHGLDCPLAFIPYCEKYR
DkTx-$(SGTx)_2$	GDCAKEGEVCSWGKKCCDLDNFYCPMEFIPHCKKYKPYVPVTTNCAKEGEVCGWGSKCCHGLDCPLAFIPYCEKYRGGGSLP ETGGGTCRYLFGGCKTTADCCKHLACRSDGKYCAWDGTFPYVPVTTTCRYLFGGCKTTADCCKHLACRSDGKYCAWDGTF

Table S2: Summary of the Kv2.1Δ7 inhibitory activity of the toxins

		**z**	**$V_{1/2}$ (mV)**	**$\Delta V_{1/2}$ (mV)**
Figure 4E	No toxin applied	3.7 ± 0.2	-11.9 ± 0.8	-
	SGTx (10 µM)	2.6 ± 0.2	26.4 ± 1.3	38.3 ± 1.5
	K1-SGTx (10 µM)	3.2 ± 0.3	4.7 ± 1.4	16.6 ± 1.6
	SGTx-K2 (10 µM)	3.2 ± 0.3	6.6 ± 1.1	18.5 ± 1.4
Figure 7B	No toxin applied	3.6 ± 0.5	-8.8 ± 1.0	-
	SGTx (3.3 µM)	2.8 ± 0.2	15.9 ± 1.3	24.7 ± 1.6
	G_3-$(SGTx)_2$ (3.3 µM)	2.9 ± 0.3	5.4 ± 1.4	14.2 ± 1.7
	DkTx-$(SGTx)_2$ (3.3 µM)	3.9 ± 0.4	4.1 ± 1.1	12.9 ± 1.5
